# Obesity and cryptorchidism across development: an integrated endocrine and metabolic life course framework

**DOI:** 10.3389/fendo.2026.1793566

**Published:** 2026-07-03

**Authors:** Dewei Zhang, Qiurong Li, Zhao Yang, Rentao Nong, Chenghao Zhanghuang, Bing Yan

**Affiliations:** 1 Department of Urology, Children’s Hospital of Kunming Medical University, Kunming Children’s Hospital, Kunming, Yunnan, China; 2 Yunnan Key Laboratory of Children’s Major Diseases Research, Yunnan Province Clinical Research Center for Children’s Health and Disease, Yunnan Clinical Medical Center for Pediatric Diseases, Kunming, Yunnan, China

**Keywords:** childhood obesity, cryptorchidism, endocrine disruptors, HPG axis, INSL3, Leydig cells, metabolic dysfunction, steroid balance

## Abstract

**Background:**

Childhood obesity is a major endocrine and metabolic disorder that extends beyond cardiometabolic risk and may affect male reproductive development. Cryptorchidism (undescended testis, UDT) is a common congenital anomaly associated with hypogonadism, subfertility, and testicular cancer. However, endocrine and metabolic links between obesity and impaired testicular descent remain incompletely integrated.

**Objective:**

This mini review synthesizes current evidence into an integrated life-course framework explaining how obesogenic exposures, including maternal metabolic disease, postnatal adiposity, and endocrine disrupting chemicals (EDCs), may interfere with testicular descent and gonadal maturation.

**Methods:**

We integrate human and experimental evidence across prenatal life, minipuberty, childhood, and puberty, focusing on Leydig cell hormones (INSL3 and testosterone), hypothalamic-pituitary-gonadal (HPG) axis regulation, sex hormone binding globulin (SHBG), aromatase activity, leptin and insulin signaling, and EDC exposure.

**Results:**

Maternal obesity and gestational metabolic disorders may be associated with an inflammatory and hormonal milieu that could plausibly impair Leydig cell function and influence testicular development. Postnatal and adolescent obesity may shift sex steroid balance through increased aromatization, reduced SHBG, and altered central gonadotropin signaling, potentially influencing gonadal maturation and testicular position maintenance. EDCs with anti-androgenic and obesogenic properties may amplify these disturbances across development.

**Conclusions:**

Integrating endocrine and metabolic mechanisms places cryptorchidism within a broader developmental and metabolic context rather than as an isolated anatomic anomaly.

## Highlights

- A life-course endocrine–metabolic lens highlights why some boys with cryptorchidism may benefit from longitudinal endocrine and metabolic surveillance.
- Obesity is associated with alterations in both central HPG-axis regulation and peripheral Leydig cell function across the life course.
- Maternal metabolic disorders, postnatal adiposity, and endocrine-disrupting chemicals converge on androgen and INSL3 signaling during critical windows of testicular descent.
- Life-course endocrine evaluation, spanning minipuberty to puberty, may contribute to improved risk stratification and long-term reproductive outcomes.
- Integrating metabolic context into cryptorchidism management supports closer endocrine–urologic collaboration and preventive strategies.

## 1 Introduction

Cryptorchidism (undescended testis, UDT) affects 2–4% of full term male infants at birth and declines to around 1% by 3 months because many testes descend spontaneously. Despite treatment, UDT is linked to reduced fertility potential and a higher risk of testicular cancer (1–3). At the same time, childhood obesity has become a global endocrine and metabolic disorder that can influence pubertal timing and gonadal hormone profiles (4, 5). These two conditions may intersect through shared endocrine mechanisms, which makes UDT relevant to endocrinology and not only to surgery.

**Figure 1:**
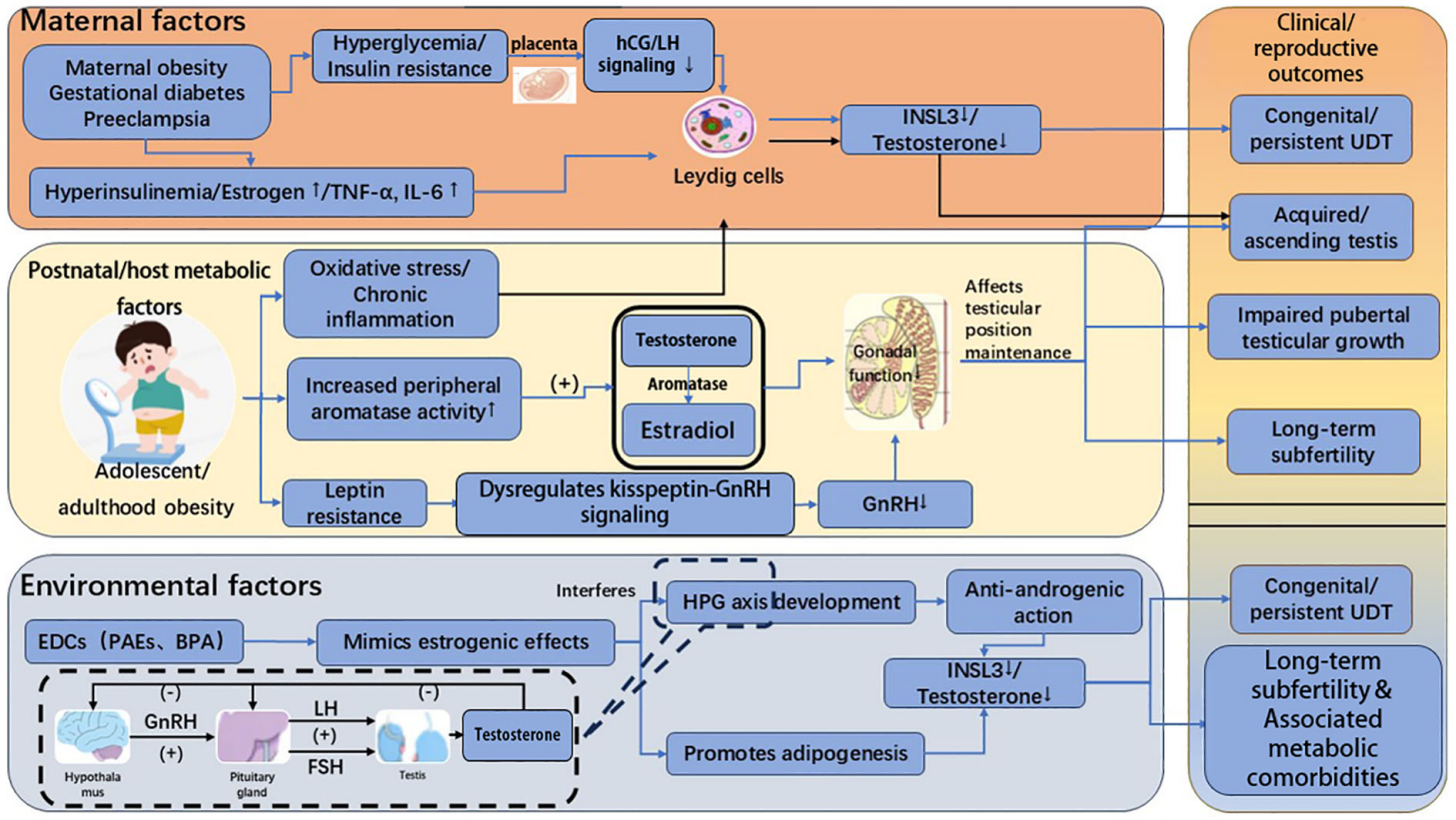
Endocrine–metabolic pathways linking obesity and cryptorchidism. Maternal obesity can promote hyperglycemia/insulin resistance and a pro-inflammatory milieu (e.g., hyperinsulinemia and increased TNF-alpha/IL-6), which may reduce fetal LH signaling and impair fetal Leydig cell output (decreased INSL3 and testosterone) during key phases of testicular descent. In postnatal life, obesity during adolescence and adulthood obesity is associated with oxidative stress/chronic inflammation, increased peripheral aromatase activity, and leptin resistance, contributing to altered sex steroid balance and reduced GnRH signaling via effects on kisspeptin neurons, thereby influencing gonadal function and testicular position maintenance. Environmental endocrine disruptors (e.g., phthalates and BPA) may mimic estrogenic effects and exert anti-androgenic actions, interfering with HPG-axis development while also promoting adipogenesis, creating a feed-forward loop between obesity and reproductive dysfunction. The resulting clinical spectrum includes congenital/persistent UDT, acquired/ascending testes, impaired pubertal testicular growth, and long-term subfertility.

Testicular descent and postnatal maturation depend on coordinated hormonal signaling across sensitive developmental windows. Obesogenic exposures may perturb androgen bioavailability, aromatization, adipokine/insulin signaling, and inflammatory tone, with endocrine-disrupting chemicals potentially compounding these effects. Because relevant evidence is dispersed across surgical, reproductive, and metabolic literatures, we summarize key mechanisms and clinical implications in a life-course framework (**Figure 1**).

Importantly, most available human data linking obesity, endocrine-disrupting chemical exposure, and cryptorchidism are observational in nature. Therefore, throughout this review, proposed endocrine–metabolic mechanisms should be interpreted as biologically plausible associations rather than established causal pathways.

## 2 Endocrine regulation of testicular descent

Testicular descent occurs in two phases: (i) the transabdominal phase, mediated by INSL3 from fetal Leydig cells acting on RXFP2 to drive gubernacular development (6, 7); and (ii) the inguinoscrotal phase, which depends primarily on androgens and genitofemoral nerve signaling (8). Variants in INSL3 or RXFP2 cause bilateral cryptorchidism, underscoring biological causality. INSL3 is a relatively stable indicator of Leydig cell function across the life course; however, its routine clinical use is currently limited by assay availability and the lack of standardized reference ranges, and it should be considered primarily a research/specialized biomarker at present (9, 10). Obesity may disrupt these pathways via reduced androgen bioavailability (low SHBG), increased adipose aromatization, and systemic inflammation that impairs Leydig steroidogenesis (**Table 1**).

**Table 1:** Endocrine pathways potentially linking obesity with impaired testicular descent and maturation.

Pathway/node	Key endocrine changes in obesity	Proposed link to cryptorchidism	Evidence strength	Candidate biomarkers/assays	Clinical/research implications
Maternal–placental inflammation	↑CRP, cytokines; placental immune activation	Impaired placental steroid handling; fetal endocrine stress → reduced androgen milieu	Human cohorts + animal/placental studies	Maternal CRP; cytokines; placental markers (research)	Target pregnancy window; prevention/intervention studies
Fetal Leydig steroidogenesis	Altered insulin/IGF, cytokines, lipid milieu	↓Steroidogenic enzyme expression/function → ↓testosterone/DHT	Animal/*in vitro*; limited human direct measures	Cord blood androgens (challenging); steroid profiles	Mechanistic placenta–testis axis studies
INSL3–RXFP2	Secondary to Leydig dysfunction	↓INSL3 output → impaired transabdominal descent	Strong genetic/animal evidence; limited obesity-specific data	INSL3 (research/selected centers)	Biomarker development; phenotype subtyping
Aromatase/estrogen excess	↑Aromatase; ↓SHBG; ↑E2 (relative)	Estrogen predominance may antagonize androgen-driven inguinoscrotal phase; suppress gonadotropins	Physiology + adolescent studies	E2, SHBG, free T calculations	Relevant for acquired/ascending testes and puberty
Leptin resistance	↑Leptin with central resistance	Impaired kisspeptin–GnRH signaling → altered LH/FSH and gonadal maturation	Experimental + clinical endocrine literature	Leptin; LH/FSH pulsatility (research)	Connects obesity to reproductive axis programming
Insulin resistance/IGF	Hyperinsulinemia; altered IGF	↓SHBG; direct Leydig effects; inflammation-mediated Leydig impairment	Human metabolic studies + mechanistic data	Fasting insulin/glucose; HOMA-IR; IGF-1	Integrate metabolic evaluation in high-risk boys
EDCs (obesogens)	Phthalates, BPA, pesticides, PFAS	Antiandrogenic/estrogenic effects; ↓steroidogenesis; ↓INSL3; concurrent obesogenic effects	Epidemiology + experimental toxicology	Urinary metabolites (phthalates/BPA)	Primary prevention; exposure window research
Genetic/epigenetic susceptibility	Variants in INSL3/RXFP2, AR pathway; epigenetic marks	Modifies sensitivity to endocrine perturbations; programs HPG axis	Genetics + emerging epigenetics	Genetic panels (selected); methylation/omics (research)	Risk stratification; precision prevention

Cryptorchidism encompasses a heterogeneous spectrum, including congenital undescended testes present at birth, persistent cryptorchidism beyond early infancy, and postnatally diagnosed forms such as ascending testes.

In this review, the term “ascending testis” is used to describe testicular ascent from a previously documented scrotal position, whereas “acquired cryptorchidism” refers to the broader clinical category of testes found outside the scrotum after a period of normal descent, which may include ascending testes.

## 3 Maternal obesity and fetal endocrine disruption

Epidemiologic studies evaluating maternal pre-pregnancy BMI and cryptorchidism have produced heterogeneous results. Some large cohort and register-based analyses suggest positive associations between maternal overweight/obesity and UDT or orchiopexy (11), whereas other well-conducted studies do not support a direct causal link (12). A meta-analysis reported that maternal diabetes during pregnancy is associated with higher cryptorchidism risk and that lifestyle factors (e.g., smoking) also contribute, while the overall effect of pre-pregnancy obesity remains inconsistent (13). These discrepancies likely reflect differences in outcome definitions (clinical diagnosis vs orchiopexy), confounding by prematurity and fetal growth, and mediation through pregnancy complications such as preeclampsia. Mechanistically, maternal obesity is characterized by insulin resistance, hyperinsulinemia, altered adipokines, and chronic low-grade inflammation that can reshape placental steroid metabolism and fetal Leydig cell function. Plausible endocrine pathways include (i) altered placental aromatase and steroid transport; (ii) inflammatory cytokines and oxidative stress impairing fetal steroidogenesis; and (iii) developmental programming of the fetal HPG axis and testicular somatic cells. Studies that jointly model maternal BMI together with metabolic mediators (glucose, insulin, lipids) and fetal/infant Leydig cell markers (testosterone and INSL3) are needed to disentangle causal structure (14). Collectively, these findings suggest that maternal metabolic inflammation and altered placental steroid handling constitute a plausible mechanistic bridge between obesity and fetal testicular dysgenesis.

## 4 Postnatal obesity and pubertal gonadal development

The neonatal period contains a transient reactivation of the HPG axis—”minipuberty”—during which basal gonadotropins and sex steroids allow functional assessment of the axis without stimulation tests (15, 16). This window is clinically important in bilateral non-palpable testes, suspected disorders of sex development, and severe genital anomalies. In boys with cryptorchidism, particularly bilateral cases, subtle disturbances of gonadotropin–testosterone dynamics and Sertoli cell markers have been reported, though findings vary by phenotype, timing, and methodology (17). Obesity may further modulate postnatal gonadal development. In adolescents and adults, obesity is associated with lower total testosterone and SHBG, relatively higher estradiol from adipose aromatization, and variable gonadotropin changes, collectively described as male obesity-related secondary hypogonadism (18–20). The interplay between adiposity, metabolic dysregulation, and gonadal function is increasingly recognized as a key mediator of reproductive disorders, with metabolic target organ damage frameworks highlighting the systemic effects of obesity on endocrine organs (21).

Pediatric data are less mature, but obesity is clearly linked to altered pubertal timing in boys (22). We propose that obesity could influence (i) pubertal testicular growth after orchiopexy via reduced androgen bioavailability; (ii) central regulation of puberty via leptin/insulin effects on kisspeptin–GnRH signaling; and (iii) risk of testicular acquired/ascending testes during childhood—a period when adiposity is increasingly common (23).

## 5 Endocrine-disrupting chemicals: anti-androgenic and obesogenic links

Endocrine disrupting chemicals (EDCs) can disrupt hormone synthesis, transport, receptor signaling, or metabolism, and several act as “obesogens” that promote adiposity through nuclear receptor pathways and metabolic reprogramming. Because testicular descent depends on Leydig cell hormones (testosterone and INSL3), anti-androgenic EDCs are plausible contributors to UDT (24, 25). A recent meta-analysis reported associations between certain prenatal EDC categories and cryptorchidism, though results differ by chemical class and study design (26). The testicular dysgenesis syndrome concept integrates UDT with other male reproductive disorders and emphasizes fetal testis vulnerability to environmental influences (27, 28). The dual anti-androgenic and obesogenic properties of some EDCs offer a coherent developmental framework for the co-occurrence of adiposity and male reproductive disorders (29).

Collectively, obesity may produce a mixed endocrine phenotype characterized by both central hypothalamic–pituitary–gonadal axis suppression and peripheral Leydig cell dysfunction. Reduced gonadotropin drive, altered sex hormone bioavailability, and impaired Leydig steroidogenesis may act synergistically across developmental windows, influencing not only testicular descent during fetal life but also postnatal testicular growth, position maintenance, and pubertal maturation.

## 6 Endocrine evaluation and follow-up implications

To translate these endocrine and metabolic concepts into clinical practice, we outline a pragmatic, scenario-based approach to assessment and follow-up in boys with cryptorchidism in the context of obesity (**Table 2**). The clinical considerations outlined below are intended to provide an endocrine-oriented conceptual framework rather than prescriptive recommendations and should be interpreted alongside existing clinical guidelines.

**Table 2:** Endocrine-oriented conceptual framework for the assessment and follow-up of cryptorchidism in the context of obesity.

Clinical scenario	Primary goals	Suggested assessments (endocrine/metabolic)	Imaging/other tests	Management considerations	Follow-up endpoints
Newborn/infant with cryptorchidism + maternal obesity history	Confirm congenital phenotype; identify high-risk endocrine presentations	Pregnancy history (BMI, gestational diabetes); if bilateral/nonpalpable: LH, FSH, testosterone (minipuberty), inhibin B/AMH; consider INSL3 where available	Imaging not routine for palpable; DSD workup if atypical genitalia	Early referral; plan orchiopexy per guideline timing; avoid routine hormonal therapy	Position; testicular volume; growth; counsel on future metabolic risk
Bilateral nonpalpable testes or suspected DSD (any BMI)	Rule out anorchia/DSD; localize testes; assess gonadal function	LH/FSH, testosterone, AMH, inhibin B; electrolytes/17-OHP as indicated; karyotype; consider hCG stimulation in specialist centers	Targeted imaging and/or diagnostic laparoscopy per protocol	Multidisciplinary endocrine–urology management; urgent evaluation	Puberty; gonadal function; fertility counseling
Childhood obesity with suspected retractile or acquired/ascending testes	Differentiate retractile vs ascending; address metabolic drivers	BMI trajectory; fasting glucose/insulin (or HOMA-IR); lipids; ALT; consider SHBG and age-appropriate testosterone/estradiol if pubertal concerns; TSH/FT4 if symptomatic	Expert physical exam; ultrasound only if exam equivocal	Lifestyle intervention; timely surgical referral if ascending/true UDT; address obesity that can hinder exam and may worsen endocrine milieu	Serial exams; testicular size; pubertal timing; cardiometabolic markers
Adolescent with obesity + delayed puberty/hypogonadal features + history of cryptorchidism	Distinguish functional HPG-axis suppression vs primary testicular injury	LH/FSH, total/free testosterone, SHBG, estradiol; prolactin/TSH as indicated; metabolic profile	Bone age if delayed; semen analysis when appropriate	Coordinate endocrine care; treat obesity; hypogonadism workup/treatment per standards	Pubertal milestones; testicular volume; semen parameters; metabolic outcomes
Syndromic obesity (e.g., Prader–Willi) with cryptorchidism	Comprehensive endocrine care + surgical correction	Syndrome-specific endocrine evaluation (GH axis, thyroid, adrenal if indicated) + gonadotropins/androgens + metabolic profile	Genetic confirmation; tailored tests as needed	Early orchiopexy; structured endocrine follow-up; family counseling	Growth, puberty, fertility counseling, metabolic health, QoL

### 6.1 Physical examination and localization in obese boys

Palpation and localization can be more challenging in obese boys. However, examination under anesthesia substantially improves accuracy, and evidence suggests that obesity does not eliminate the utility of careful physical examination in experienced hands (30). Endocrine–urology teams should document testis position (high scrotal vs inguinal vs non-palpable), laterality, and associated genital anomalies.

### 6.2 Orchiopexy timing and endocrine windows

Current guidelines recommend orchiopexy for persistent UDT within the first year of life to optimize fertility potential (31–33). From an endocrine perspective, early correction aligns with periods of active germ cell maturation and may reduce heat-related injury.

### 6.3 Endocrine work-up and metabolic screening

Bilateral non-palpable testes, micropenis, hypospadias, or syndromic features warrant endocrine evaluation—ideally during minipuberty— (2, 16). Suggested biomarkers include LH/FSH, testosterone, AMH, inhibin B, and where available INSL3 (9, 10). In obese children with UDT or suspected ascent plus growth/puberty concerns, metabolic screening (glucose/insulin, lipids, liver enzymes) can be considered as part of a broader endocrine assessment.

### 6.4 Pubertal follow-up

For adolescents after orchiopexy—especially those with obesity—follow-up can focus on testicular volume, pubertal progression, and hormone profiles when clinically indicated. Total testosterone should be interpreted in the context of SHBG; measuring free testosterone or calculating it may be useful in obesity where SHBG is low (19, 20). Follow-up may reasonably extend into adolescence and adulthood, particularly in selected high-risk contexts.

## 7 Knowledge gaps and future directions

Future studies should move beyond cross-sectional associations and adopt longitudinal, life-course designs. First, prospective cohorts are needed to characterize trajectories of LH/FSH, testosterone, estradiol, SHBG, inhibin B, AMH, and INSL3 from minipuberty through late puberty, with stratification by adiposity status and cryptorchidism phenotype. Recent evidence on physiological and disordered minipuberty, together with cohort-based reference data for infant reproductive hormones, provides an important methodological basis for such work (34, 35). Second, causal inference approaches should be used to disentangle the contribution of maternal BMI from related metabolic mediators, including gestational diabetes, preeclampsia, insulin resistance, inflammatory status, and concurrent endocrine-disrupting chemical exposure (36, 37). Third, mechanistic studies should clarify how adipokine, insulin, inflammatory, and lipid-related signals affect fetal Leydig cell steroidogenesis, INSL3 production, and gubernacular development during critical windows of testicular descent. Finally, preventive studies are needed to determine whether optimization of maternal metabolic health or reduction of endocrine-disrupting chemical exposure can modify the risk of UDT or improve long-term reproductive outcomes. Longitudinal mother–son cohort studies linking prenatal environmental exposure to adult testicular endocrine function provide a useful model for this research direction, although replication in pediatric and cryptorchidism-specific populations remains necessary (38).

Harmonized phenotyping will be essential, particularly the distinction between congenital/persistent UDT, retractile testes, and acquired or ascending testes. Integration of biobanks, birth cohorts, environmental exposure datasets, and registry-based surgical follow-up may help define clinically meaningful endocrine–metabolic subgroups. Importantly, the population-level contribution of obesity-related endocrine perturbations to cryptorchidism remains uncertain. Current evidence supports the view that maternal and postnatal metabolic factors are more likely to act as modifiers of susceptibility rather than as primary determinants of testicular maldescent. Therefore, the framework proposed in this review should be interpreted as a hypothesis-generating model that can guide future longitudinal, mechanistic, and interventional studies at the interface of pediatric endocrinology and urology.

Several mechanisms discussed in this review are derived from adult, experimental, or non-pediatric studies. Their applicability to children, especially across distinct cryptorchidism phenotypes, requires cautious interpretation and pediatric-specific validation.

## 8 Conclusions

Cryptorchidism and obesity intersect through shared endocrine and metabolic pathways operating across sensitive developmental windows. Rather than implying direct causality, current evidence supports a model in which metabolic dysregulation modifies hormonal environments critical for testicular descent and postnatal gonadal maturation.

Viewing cryptorchidism through an endocrine–metabolic lens reframes it as a developmental condition influenced by systemic metabolic context rather than a purely anatomical anomaly. This perspective highlights the value of life-course assessment and interdisciplinary collaboration, while underscoring the need for longitudinal studies to define clinical relevance and effect size more precisely.
